# Migraine is associated with an increased risk for benign paroxysmal positional vertigo: a nationwide population-based study

**DOI:** 10.1186/s10194-015-0547-z

**Published:** 2015-07-04

**Authors:** Chia-Huei Chu, Chia-Jen Liu, Liang-Yu Lin, Tzeng-Ji Chen, Shuu-Jiun Wang

**Affiliations:** Department of Otorhinolaryngology-Head and Neck Surgery, Taipei Veterans General Hospital, Taipei, Taiwan; Division of Hematology and Oncology, Department of Medicine, Taipei Veterans General Hospital, Taipei, Taiwan; Division of Endocrinology and Metabolism, Department of Medicine, Taipei Veterans General Hospital, Taipei, Taiwan; Department of Family Medicine, Taipei Veterans General Hospital, Taipei, Taiwan; Department of Neurology, Neurological Institute, Taipei Veterans General Hospital, Taipei, Taiwan; Department of Otorhinolaryngology, National Yang-Ming University School of Medicine, Taipei, Taiwan; Institute of Public Health, National Yang-Ming University School of Medicine, Taipei, Taiwan; Department of Medicine, National Yang-Ming University School of Medicine, Taipei, Taiwan; Department of Neurology, National Yang-Ming University School of Medicine, Taipei, Taiwan; Institute of Brain Science, National Yang-Ming University School of Medicine, Taipei, Taiwan; Brain Research Center, National Yang-Ming University School of Medicine, Taipei, Taiwan

**Keywords:** Migraine, Benign paroxysmal positional vertigo, Risk factors, Incidence rate

## Abstract

**Background:**

There is evidence suggesting that migraine may be associated with vertigo. The aim of this study was to assess the risk of benign paroxysmal positional vertigo (BPPV), the most common form of vertigo, in patients with migraine using a population-based dataset.

**Methods:**

The National Health Insurance Research Database in Taiwan was searched for migraine patients and was also used to select an age- and sex-matched cohort of subjects without migraine. The analyses included 8266 migraine patients and 8266 controls. The incidence rates of BPPV in the two cohorts were compared. Cox proportional hazard models were used to identify risk factors for BPPV in migraine patients.

**Results:**

In the migraine cohort, 1.11 % of the patients developed BPPV compared to 0.5 % of the controls. The incidence rate ratio was 2.03 (95 % CI 1.41–2.97; p <0.001). Cox proportional hazards analysis showed that age ≥40 years (HR 2.20; 95 % CI 1.40–3.45; *p* = 0.001), coronary artery disease (HR 4.62; 95 % CI 1.12–19.01; *p* = 0.034), and the number of outpatient department visits to neurologists because of migraine (HR 2.93; 95 % CI 2.50–3.44; p >0.001) were associated with an increased risk for BPPV.

**Conclusion:**

The results showed that patients with migraine had a 2.03-fold increased risk of developing BPPV compared with age- and sex-matched controls. Although BPPV may not be a common condition in migraine patients, migraine sufferers with vestibular symptoms should alert physicians to the possibility of BPPV, particularly if patients are aged ≥40 years, have a history of coronary artery disease, or have frequent visits to neurologists clinics because of migraine.

## Background

Both migraine and vertigo rank among the most common complaints in the general population. Making an association between migraine and dizziness dates back to the 19th century when Liveing first mentioned their connection [1]. Later, a Kayan and Hood reported that migraine patients had a much higher incidence of vertigo than the tension headache patients (26.5 % versus 7.8 %) [2]. An epidemiological study had reported that there might be a link between migraine and vertigo [3]. In Taiwan, migraine has a prevalence ranging from 8 % to 15 % [4]. People between the ages of 25 and 55, usually the most productive years of life, have the highest prevalence of migraine [5].

A number of studies have reported findings which suggesting that repeated migraine attacks can result in progressive structural and functional alterations of the brain [6]. Migraine also is a risk factor for ischemic stroke and there are reports of ischemic stroke occurring during migraine attacks [6, 7].

The prevalence of dizziness and vertigo in the general population is approximately 20 % to 30 % [3]. Many migraine patients suffered from dizziness or vertigo. Benign paroxysmal positional vertigo (BPPV) is the most frequent cause of vertigo [8]. It can be easily diagnosed and usually rapidly corrected. Studies have reported that the prevalence ranges from 10.7 to 64.0 per 100,000 population [8] with a life time prevalence of 2.4 %. The prevalence is increased among elderly persons and among women, with peak onset between 50 and 60 years of age and a female-to-male ratio of 2:1 to 3:1 [9]. BPPV is characterized by brief spinning sensations lasting less than 1 min, which are generally induced by an abrupt change in head position with respect to gravity. It is believed to be caused by otolith, i.e., small calcium carbonate crystals that enter the semicircular canals from the utricle, one of the otolith organs in the inner ear. A change of the head position results in these crystals travelling within the semicircular canals which drives movement of the endolymph causing the sensation of vertigo [8]. Clinically, BPPV can usually be diagnosed based on the nystagmus induced by Dix-Hallpike maneuver [8, 10] and can often be successfully treated immediately using Epley’s canalith-repositioning maneuver [8].

Previous researches have shown that migraine and vestibular dysfunction may have potential interactions and relationships via several conditions including BPPV or other disorders related to inner ear dysfunction [11, 12]. Therefore, the aim of this study was to explore the risk of BPPV in migraine patients.

## Materials and methods

### Data sources

Taiwan launched the National Health Insurance (NHI) program in 1995 to finance health care for all citizens. The NHI system now covers 99.9 % of Taiwan’s population, and 93 % of the country’s hospitals and clinics are NHI-contracted [13]. The NHI is a mandatory, single-payer social insurance system, offering almost universal medical care. It includes coverage for outpatient, inpatient, emergency, dental, traditional Chinese medicine services, and prescription drugs. In 1999, the Bureau of National Health Insurance made patient data available electronically through the National Health Insurance Research Database (NHIRD) project. The multiple NHI databases (i.e. NHI enrollment files, claims data, and registry of drug prescriptions) provide comprehensive utilization and enrollment information for all patients under the NHI program. This study used NHI databases that are managed and publicly released by the National Health Research Institute of Taiwan. The confidentiality of the data abides by the regulations of the Bureau of the NHI and the National Health Research Institute. All information that would potentially expose a specific individual to be identified has been encrypted. Since the NHI dataset consists of de-identified secondary data for research purposes, this study was exempt from full review by the institutional review board.

### Study population

A retrospective cohort study was conducted using a dataset from January 1, 2000 to December 31, 2009. Patients who were diagnosed with migraine by a neurologist according to the migraine diagnosis codes (346.0×, 346.1×, and 346.9×) in the International Classification of Diseases, Ninth Revision, Clinical Modification (ICD-9-CM) were enrolled. Patients diagnosed with migraine at an age < 20 or ≥ 65 years were excluded. Patients with antecedent BPPV (386.11) within one year before the diagnosis of migraine were also excluded. In addition, those diagnosed with acoustic neuroma (225.1), Meniere’s disease (386.0×), vestibular neuritis (386.12), labyrinthitis (386.3×), sudden hearing loss (388.2), or head injury (310.2, 800.×-804.×, 850.×-854.×, 870.×-873.×, 907.0, 907.1, 959.0×, and V15.52) at any time within the study period were also excluded from the analysis. Subjects without migraine were randomly selected from the same database for the matched (non-exposure) cohort. Each migraine patient was matched with one non-exposure subject by age and sex within the same observational period.

Both the migraine cohort (*n* = 8,266) and the matched cohort (*n* = 8,266) were followed up until the development of BPPV, transfer out of the NHI program, death, or the end of the year 2010.

### Outcome

The outcome variable of this study was new occurrence of BPPV, as defined by a compatible ICD-9-CM code 386.11 that was coded by a neurologist or an otorhinolaryngologist.

#### Statistical analysis

Categorical variables were presented as counts and percentages, and the chi-square test was performed for group comparisons. The incidence rates (per 100,000 person-years) with 95 % confidence intervals (CIs) and incidence rate ratios (IRRs) of BPPV in both groups were analyzed. The Kaplan-Meier method and the Cox proportional hazard models were used to explore the associations between each possible confounding factor and BPPV. Factors with *p* < 0.1 in univariate analyses were then included in a multivariable model. Extraction and computation of data were performed with the Perl programming language (version 5.12.2). A Microsoft SQL Server 2005 (Microsoft Corp., Redmond, WA, USA) was used for data linkage, processing, and sampling. The statistical analyses were carried out with IBM SPSS statistical software version 19.0 for Windows (IBM Corp., Armonk, New York, USA). Statistical significance was defined as a *p* value less than 0.05.

## Results

### Demographic characteristics of the study population

During the 10-year study period, a total of 14,164 migraine patients were diagnosed or had diagnoses confirmed by neurologists. After excluding patients with aged < 20 (*n* = 1,255) or ≥ 65 years (*n* = 1,304), BPPV before migraine diagnosis (*n* = 65), acoustic neuroma (*n* = 21), Meniere’s disease (*n* = 789), vestibular neuritis (*n* = 372), labyrinthitis (*n* = 38), sudden hearing loss (*n* = 35), and head injury (*n* = 2,019) diagnosed at any time within the study period, the final sample consisted of 8266 patients for the analyses.

The demographic characteristics of the migraine cohort and matched cohort are presented in Table 1. In each cohort, the median age was 38 years and 72 % of the cohort were females. Among the migraine patients, 8.93 % were diagnosed as having migraine with aura. No significant differences were found between the migraine cohort and matched cohort in comorbidities (Table 1). The median follow-up duration was 6.24 years for the matched cohort and 6.29 years for the migraine cohort.

**Table 1:** Baseline characteristics of patients with migraine and matched cohort

Characteristics	Migraine cohort		Matched cohort		*P* value
	*n* =8266		*n* =8266		
	No.	%	No.	%	
Median age, years (interquartile range)	38 (29–48)		38 (29–48)		
Age, years					
<40	4408	53.3	4408	53.3	1.000
≥40	3858	46.7	3858	46.7	
Sex					
Female	5952	72.0	5952	72.0	1.000
Male	2314	28.0	2314	28.0	
Comorbidities					
Diabetes mellitus	811	9.8	810	9.8	0.979
Hypertension	1460	17.7	1460	17.7	1.000
Heart failure	159	1.9	152	1.8	0.689
COPD	1081	13.1	1079	13.1	0.963
Asthma	873	10.6	870	10.5	0.939
Chronic kidney disease	541	6.5	539	6.5	0.950
Coronary artery disease	35	0.4	29	0.4	0.452
Dyslipidemia	1429	17.3	1429	17.3	1.000
Cirrhosis	67	0.8	58	0.7	0.419
Autoimmune diseases	588	7.1	582	7.0	0.856
Cerebrovascular disease	876	10.6	869	10.5	0.859
Previous BPPV	69	0.8	58	0.7	0.327
Migraine with aura	738	8.93			

*Abbreviations:* *COPD* chronic obstructive pulmonary disease, *BPPV* benign paroxysmal positional vertigo

### Comparisons of the incidence rates of BPPV between migraine patients and the matched cohort

Comparisons of BPPV incidence rates between the migraine patients and the matched control group are presented in Table 2.

**Table 2:** The risk of benign paroxysmal positional vertigo (BPPV) for patients with migraine and matched cohort

Characteristics	Patients with migraine, *n* = 8266		Matched cohort, *n* = 8266		IRR (95 % CI)	*P* value
	BPPV No.	Per 10,000 person-year	BPPV No.	Per 10,000 person-year		
Total	92	18.1	45	8.9	2.03 (1.41–2.97)	<0.001
Age						
<40	29	10.6	11	4.0	2.63 (1.28–5.84)	0.004
≥40	63	26.8	34	14.6	1.84 (1.19–2.88)	0.004
Sex						
Male	20	14.1	12	8.5	1.66 (0.77–3.72)	0.168
Female	72	19.7	33	9.1	2.17 (1.42–3.39)	<0.001
Aura						
With aura	10	24.2	4	9.8	2.48 (0.71–10.81)	0.123
Without aura	82	17.5	41	8.8	1.99 (1.35–2.97)	<0.001

*Abbreviations:* *IRR* incidence rate ratio, *CI* confidence interval

Chi-square tests were used for the group comparisons

Ninety-two (1.11 %) patients in the migraine cohort and 45 (0.5 %) subjects in the matched cohort developed BPPV (Fisher’s exact test, *p* < 0.001). The migraine cohort had a greater risk of developing BPPV than the matched cohort (IRR = 2.03; 95 % CI 1.41–2.97, p < 0.001). Both the older (aged ≥ 40 years) and the younger (age < 40 years) patients with migraine had a higher incidence of BPPV than the matched controls (26.8 vs. 14.6 per 100,000 person-years and 10.6 vs. 4.0 per 100,000 person-years, *p =* 0.004, respectively). The female migraine subgroup had a higher IRR than the male subgroup, and this reached statistical significance in the female subgroup (19.7 versus 14.1 per 100,000 person-years, Fisher’s exact test, *p* < 0.001). Patients diagnosed as having migraine without aura had a significantly higher IRR compared with the matched controls (IRR = 1.99, 95 % CI 1.35–2.97, *p* < 0.001). Only 10 of the 738 patients who had migraine with aura developed BPPV, and no significant difference in IRR was found between the migraine patients and controls (IRR = 2.48, 95 % CI 0.71–10.81, *p* = 0.123) (Table 2).

**Fig. 1:**
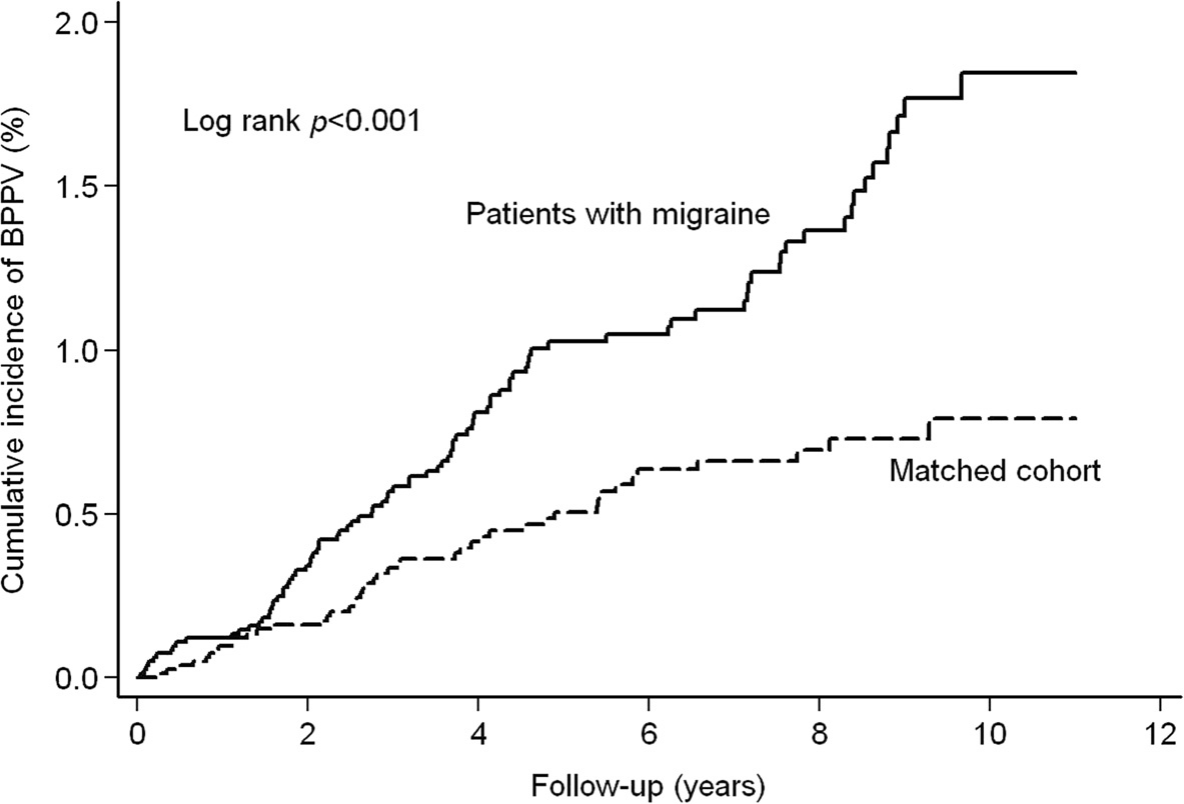
Cumulative incidence of benign paroxysmal positional vertigo in migraine and matched cohort

The cumulative incidence of BPPV in patients with migraine was significantly higher than that in the matched cohort (log rank, *p* < 0.001, Fig. 1).

### Risks factors for BPPV in migraine patients

Univariate Cox regression analysis revealed a significantly increased risk of BPPV in migraine patients with the following characteristics: age ≥40 years, hypertension, chronic obstructive pulmonary disease (COPD), coronary artery disease (CAD), cerebrovascular disease, and number of outpatient department (OPD) visits to see a neurologist for migraine (all p < 0.05). If the factors in univariate analysis have a *p*-value ≤ 0.1, they were included in multivariate analysis. The results of multivariable Cox proportional hazards analysis showed there was a significantly higher hazard risk of developing BPPV in the migraine cohort associated with age ≥40 years (HR = 2.2, *p* = 0.001), CAD (HR = 4.62, *p* = 0.034), and the number of visits to neurologist’s OPD for migraine (HR = 2.93, *p* < 0.001) (Table 3).

**Table 3:** Risk factors for benign paroxysmal positional vertigo in patients with migraine

	Univariate analysis			Multivariable analysis^a^	
	Univariate analysis			Multivariable analysis^a^	
Variables	HR (95 % CI)	*P* value		HR (95 % CI)	*P* value
Age ≥ 40 years	2.53 (1.63–3.92)	<0.001		2.20 (1.40–3.45)	0.001
Male sex	0.72 (0.44–1.17)	0.186			
Comorbidities					
Diabetes mellitus	0.58 (0.24–1.42)	0.233			
Hypertension	1.97 (1.25–3.1)	0.003			
Heart failure	2.00 (0.63–6.32)	0.238			
COPD	1.93 (1.16–3.20)	0.011			
Asthma	1.34 (0.71–2.51)	0.366			
Chronic kidney disease	1.33 (0.62–2.88)	0.467			
Coronary artery disease	6.35 (1.56–25.8)	0.010		4.62 (1.12–19.01)	0.034
Dyslipidemia	1.56 (0.95–2.56)	0.080			
Cirrhosis	2.78 (0.69–11.3)	0.152			
Autoimmune diseases	1.64 (0.82–3.27)	0.159			
Cerebrovascular disease	2.60 (1.60–4.22)	<0.001			
Previous BPPV	3.54 (0.87–14.39)	0.077			
Neurologists’ OPD visit for migraine	3.00 (2.56–3.51)	<0.001		2.93 (2.50–3.44)	<0.001
Migraine with aura	1.38 (0.71–2.65)	0.340			

*Abbreviations: HR* hazard ratio, *CI* confidence interval *COPD* chronic obstructive pulmonary disease, *BPPV* benign paroxysmal positional vertigo

^a^All factors with *p* < 0.1 in univariate analyses were selected for Cox multivariable analysis

The reference groups for each variable were as follows: Age < 40 years, female, without co-morbidities, without neurologist OPD visit, or without aura, respectively

## Discussion

This nationwide, population-based, cohort study demonstrated that migraine was associated with an increased risk of developing BPPV that was 2.03 times higher than that of the matched controls. The incidence rate was 18.1 per 100,000 person-years in the migraine cohort in contrast to 8.9 in the matched control cohort. Furthermore, there was a higher risk of developing BPPV associated with three factors: migraineurs aged ≥40 years (HR =2.20), CAD (HR =4.62) as well as neurologists’ OPD visit. To the best of our knowledge, this research is the first population-based study to demonstrate that migraine is associated with an increased risk for BPPV.

Comorbid conditions such as hypertension, coronary artery disease, and cerebrovascular disease would be expected to be more common in a non-migraine population than in a non-migraine population, however, in this study there were no significant differences in prevalence of comorbid conditions between the migraine group and non-migraine control group. The reason for this was that not only were the patients in the migraine group and control group perfectly matched for sex and age but we also tried to match the groups for comorbid conditions (Table 1).

There is now considerable epidemiological evidence linking migraine to vertigo as well as dizziness. Among patients whose are diagnosed as having migraine, about 25 % also complain of having vertigo and about 30 % complain of dizziness [14]. In studies of patients with dizziness, it has been found that such patients have a higher prevalence of migraine than would be expected and in researches of patients with migraine it has been found that such patients have an increased prevalence of vertigo and dizziness [3]. A recent cross-sectional study by Calhoun et al investigated the point prevalence of dizziness or vertigo in patients with migraine [15]. They found that patients who had migraine with aura had a prevalence of dizziness or vertigo that was twice as high (24.5 % versus 12.1 %) as patients with migraine without aura and that the prevalence of dizziness or vertigo was significantly associated increased age. They also reported a significant relationship between severity of migraine pain and increased complaints of vertigo. Almost half of the migraineurs reported concomitant dizziness or vertigo when migraine pain was present at an intensity of 7 or greater (on a scale of 1–10). In our study, age ≥40 years was noted to be a risk factor for BPPV in patients with migraine, and this finding was also presented in our study. Warninghoff et al carried out the first study to investigate systematically the co-morbidities of vertiginous diseases [16]. In this survey study of 131 participants with various kinds of vertiginous diseases, no increased prevalence (9.4 %) of migraine was found compared with an epidemiological study of a 6-month prevalence 11.2 % for migraine. The relatively small sample size may explain the finding. A retrospective study of 476 patients with BPPV conducted by Uneri revealed that migraine and motion sickness were three times more common in patients with BPPV than in the general population and a family history of migraine (58.4 %) was also more common [17]. Another research by Fararilli et al investigated 186 BPPV in patients with migraine [18]. They compared a group of patients with BPPV and migraine with a group of patients with BPPV but without migraine or other type of headache. They found that mean age of BPPV onset was earlier in the former group (39 versus 53 years) and highly recurrent BPPV occurred more often in the former group (19.4 % versus 7.3 %). The findings suggested that the clinical features of BPPV are affected by migraine.

The major strength of this study was its population-based design, which examined a representative cohort of 1 million citizens covered under the NHI in Taiwan. The large sample size and long observation period offered enough power to delineate the differences between the two study groups. To achieve a high validity, the enrolled migraine patients had to be diagnosed only by neurologists, and the outcome variable, new onset BPPV, had to be coded by a neurologist or an otorhinolaryngologist. Additionally, patients with certain common inner ear diseases such as Meniere’s disease, acoustic neuroma, vestibular neuritis, labyrinthitis, and sudden hearing loss, and those who had head injury at any time within the study period were excluded since BPPV might be related or secondary to these disorders. The strict inclusion and exclusion criteria minimized possible coding deviations in this claims dataset.

No direct pathophysiological link between migraine and BPPV has yet been established. Baloh in 1997 had postulated that neuro-otological symptoms in migraine patients may originate from vasospasm and/or a certain ion channel disorder [1]. Ishiyama et al once hypothesized that patients with migraine suffered recurrent damage to the inner ear because of vasospasm or some other mechanism, which predisposes them to episodes of BPPV [19, 20]. Therefore it could be inferred that repetitive vasospasms or disturbance of vestibule-cochlear microvasculature might play a role in inner ear insult, resulting in damages of the epithelium in the vestibule, causing dislodge of the otoconia from utricular macula into semicircular canal, and thus give rise to BPPV. The same postulation may also explain the correlation between migraine and various hearing problems that had been largely reported [21, 22]. It should also be noted that a neurogenic inflammation in the inner ear in experimental migraine animal models has been reported [23]. Such recurrent neurogenic inflammation might accelerate degeneration of the otoliths organ. However it remains unclear what triggers this inflammation.

Various specific chemical mediators such as nitric oxide, serotonin receptors, calcitonin gene-related peptide, and prostanoids have been found to play a role in the headache phase of a typical migraine attack. And in the postdromal phase, in which some migraine symptoms persist after the headache ends, is characterized by persistent blood flow changes in certain regions of the brain [20]. In our study, CAD was associated with an increased risk (HR 4.62) for BPPV in migraineurs. This finding could possibly echo the assumption that vasospasm and the downstream vascular events that take place intracranially as well as in the inner ear may at least partially explain the relationship between migraine and BPPV. Further investigations would be needed to clarify the underlying causes for the link between migraine and BPPV.

Our study has a number of clinical implications. Physicians must be more alert to vestibular complaints of migraine patients, especially in those older than 40 years, with CAD, or who seek frequent OPD help for migraine, since they are more likely to develop BPPV. Early recognition and prompt management of BPPV are important in resolving patients’ discomforts. Finally, migraine history should be routinely inquired about in patients with BPPV. Examining migraine history can be used to clarify the etiology and to delineate whether BPPV development in migraineurs could have a different clinical pattern or prognosis.

The followings are limitations of this study. First, deviations in coding of the claims data were inevitable. A matched control cohort was used to offset the possible coding errors and to minimize bias. The Bureau of the NHI routinely and randomly samples a fixed percentage of claims from every contracted medical institution, and the accuracy of coding is audited by an independent group of physicians [13, 24, 25]. Second, it is difficult to explore the prognosis of BPPV, since the NHIR database is an administrative database that lacks detailed clinical data on the severity and outcome of BPPV. Third, the small sample size of certain subgroups might have contributed to failure in showing migraine as a significant risk factor in the subgroup analyses. Fourth, a major differential diagnosis is central positional vertigo due to a lesion in the cerebellum and its connection to the vestibular nuclei, and in addition, positional nystagmus can sometimes be seen during vestibular migraine attacks. However, in this study we tried to achieve high validity by obtaining coding from a neurologist or an otorhinolaryngologist. Therefore, we believe this considerably improved the quality of coding.

## Conclusions

In conclusion, our study demonstrated that patients with migraine had a 2.03-fold greater risk of developing BPPV compared to the age- and sex-matched control cohort. Although BPPV is a rare condition, vestibular symptoms in migraine patients should still alert physicians to the possibility of occurrence of BPPV, especially those who are older than 40, have CAD, or who make frequent OPD visits for migraine.

## Abbreviations

BPPV: Benign paroxysmal positional vertigo
CAD: Coronary artery disease
CIs: Confidence intervals
COPD: Chronic obstructive pulmonary disease
HR: Hazard ratio
ICD-9-CM: International Classification of Diseases, Ninth Revision, Clinical Modification
IRRs: Incidence rate ratios
NHI: National Health Insurance
NHRI: National Health Research Institute
OPD: Outpatient department
SSNHL: Sudden sensorineural hearing loss
